# Evolution of Hair Treatment and Care: Prospects of Nanotube-Based Formulations

**DOI:** 10.3390/nano9060903

**Published:** 2019-06-21

**Authors:** Ana Cláudia Santos, Abhishek Panchal, Naureen Rahman, Miguel Pereira-Silva, Irina Pereira, Francisco Veiga, Yuri Lvov

**Affiliations:** 1 Department of Pharmaceutical Technology, Faculty of Pharmacy, University of Coimbra, Azinhaga Sta. Comba, 3000-548 Coimbra, Portugal; acsantos@ff.uc.pt (A.C.S.); miguelsp_20@hotmail.com (M.P.-S.); irina.pereira@live.com.pt (I.P.); fveiga@ci.uc.pt (F.V.); 2 REQUIMTE/LAQV, Department of Pharmaceutical Technology, Faculty of Pharmacy, University of Coimbra, 3000-548 Coimbra, Portugal; 3 Institute for Micromanufacturing, Louisiana Tech University, P.O. Box 10137, Ruston, LA 71272, USA; agp010@latech.edu (A.P.); nra015@latech.edu (N.R.); 4 Theoretical Physics & Applied Mathematics Department, Ural Federal University, 620002 Ekaterinburg, Russia

**Keywords:** hair treatment, halloysite clay nanotubes, hair dyeing, drug sustained release

## Abstract

A new approach for hair treatment through coating with nanotubes loaded with drugs or dyes for coloring is suggested. This coating is produced by nanotube self-assembly, resulting in stable 2–3 µm thick layers. For medical treatment such formulations allow for sustained long-lasting drug delivery directly on the hair surface, also enhanced in the cuticle openings. For coloring, this process allows avoiding a direct hair contact with dye encased inside the clay nanotubes and provides a possibility to load water insoluble dyes from an organic solvent, store the formulation for a long time in dried form, and then apply to hair as an aqueous nanotube suspension. The described technique works with human and other mammal hairs and halloysite nanoclay coating is resilient against multiple shampoo washing. The most promising, halloysite tubule clay, is a biocompatible natural material which may be loaded with basic red, blue, and yellow dyes for optimized hair color, and also with drugs (e.g., antilice care-permethrin) to enhance the treatment efficiency with sustained release. This functionalized nanotube coating may have applications in human medical and beauty formulations, as well as veterinary applications.

## 1. Introduction

Beauty is inherent to human wellbeing and hair has aesthetic, historic, and cultural significance, and is also an indicator of health. Each hair is a skin appendage that consists of a root embedded in the dermis and a hair shaft, a flexible strand composed of hard keratin fibers (rich in cysteine) bound through strong (disulfide) and weak (hydrogen) bonds. In the dermis, the hair root is surrounded by the hair follicle, an epidermal invagination. The basis of the hair follicle is a bulb. In the hair shaft cross-section, three essential constituents are identified: the inside medulla, circumvent by the cortex, which is overlaid by the cuticle, the outermost layer of the hair. [1,2]. The cortex is composed of elongated keratin-rich cortical cells with a unique microstructure responsible for the strength and elasticity, texture, and healthy visual appearance of the hair. Melanin, produced by melanocytes, gives the hair color and is found in the cortex of the hair shaft. Two types of melanin can be distinguished: eumelanin (confers darker tones) and pheomelanin (confers lighter tones). The final hair color is a mixture of these two types of melanin [3]. At older ages, melanin production is diminished, as well as the number of melanocytes, and hair gradually turns grey [4]. The cuticle is the hair’s protective barrier, formed by multiple layers of keratinized, flattened cells (scales), which overlap in a roof-tile formation [1,2]. Nearly the entire surface of the human body is covered by hair, but the majority (above 100,000) of hair follicles exist on the scalp [5].

Hair care is an area of the cosmetic and pharmaceutical industries, which invest in formulations for appearance (hair color) and treatment of scalp and hair diseases (e.g., pediculosis and dandruff). Hair care cosmetic formulations include shampoos, conditioners, styling products (gels, waxes, and mousses), and hair dyes. Surfactants that are anionic, amphoteric, or non-ionic are shampoo cleaning agents. Conditioners needed to neutralize an excessive negative charge of washed hair and silicones are the most common additives [6]. Any newly suggested hair color formulations have to be compatible with these components.

There are permanent, semi-permanent, and temporary dyes, depending on the molecular structure and the cuticle penetration capabilities [7]. Permanent dye colors have aromatic amines (para-phenylenediamine) and couplers (e.g., resorcinol and meta-aminophenols) in the presence of a strong oxidant (hydrogen peroxide) and an alkalinizing agent (ammonia). A multitude of colors can be created, based on blue, red, and yellow-green couplers [2]. Temporary dye molecules do not penetrate the cuticle layer, and are adhered to the external surface by weak forces [8]. Safe, naturally-occurring dyes can be found in fruits (juglone–5-hydroxy-1,4-naphthoquinone in walnuts) and plants like *Indigofera* ((2E)-2-(3-oxo-1H-indol-2-ylidene)-1H-indol-3-one). Henna is a natural red dye obtained from the plant *Lawsonia alba*, by extracting lawsone (2-hydroxy-1,4-naphthoquinone) [9,10]. Red henna, blue indigo with the addition of a yellow curcumin mixture, delivers brown colors that have been commonly applied on grey hair for centuries.

Hair coloring formulations have to be examined for their toxicity, ranging from allergic contact dermatitis and skin irritations, to carcinogenicity [11]. The high alkaline pH and hydrogen peroxide in oxidative dyes contribute to damage of the hair shaft and often cause allergies. Another concern is that irritating dye molecules reach the scalp and face skin. Hair dye application from neutral water solutions, avoiding chemical reactions during coloring, could be a promising strategy for new formulations. We will demonstrate such hair coloring methods based on a self-assembly coating of pigment clay nanotubes from aqueous dispersion.

Permanent hair dyes often have a negative effect on biological organisms by triggering allergic responses, such as contact dermatitis [3]. Nano/micro encasings could decrease dyes toxicity, e.g., the polyglutamic acid encapsulated polydiacetylate allowed for 200–300 nm particles, which were applied for safe hair coloring [12].

Dermatological afflictions, such as hair loss, affect a large amount of the population and have been a subject of intensive research. For better delivery, nanostructured lipid carriers were developed with minoxidil and finasteride, showing a synergic action of the two anti-alopecia used drugs [13]. Polymeric nanoparticles were synthesized for finasteride topical delivery into hair follicles [14]. Results showed a reduced finasteride skin penetration, enhancing its permanence time and durability of therapeutic effect, as well as a prolonged release for over 3 h.

A synthesis of gold nanoparticles inside the hair cortex showed brown color for 16 days. Chloroauric acid in alkaline solvent formed 10–20 nm gold particles with HAuCl_4_ reduction. Amino acid-cysteine enhanced this reduction, and the majority of gold nanoparticles occurred in the keratin regions, enriched with cysteine [15]. Melanin extracted from cuttlefish ink was encapsulated in tiny lipid capsules for hair delivery via micro-needling inside the follicles, thus darkening grey hair [16]. These hair-delivered nanoparticles allowed deeper penetration into the follicles and increased color storage [17,18]. The idea of encapsulation of dyes into nanoscale tubular containers capable of being assembled on hair looks very promising and has already produced some results.

Carbon materials, particularly graphene-based nano-sheets, have also shown promise for hair dyeing. Graphene oxide is dispersible in water and may be safely used in cosmetics, contrary to carbon nanotubes. Graphene oxide hair formulations were mixed with chitosan, giving brown to black colors. They demonstrated a durable effect, resisting multiple shampooing, antistatic, and heat dissipation [19]. Fullerene nanoparticles have also been explored for hair growth stimulation [20]. Finasteride encapsulated in liquid crystalline nanoparticles gave a decrease in posology and unwanted side effects due to scalp skin retention [21].

Nanotubes, nanoscale materials with a tubular shape, are promising for dye encapsulation, producing versatile and stable nanopigments [22]. Despite the many engineering applications of carbon nanotubes, they are unsafe for health care and other types of nanotubes are more promising due to their non-toxic aqueous processing. They are naturally-occurring halloysite clay nanotubes (HNT), silica nanotubes, boron-nitride nanotubes, and nickel vanadate nanotubes. We developed versatile nanoclay-based materials for health and personal care applications [23]. We emphasize a potential usage of biocompatible and abundantly available halloysite clay nanotubes for haircare formulations.

## 2. Nanotube-Based Formulations for Human Hair Treatment

### 2.1. Halloysite Clay Nanotubes

**Scheme 1:**
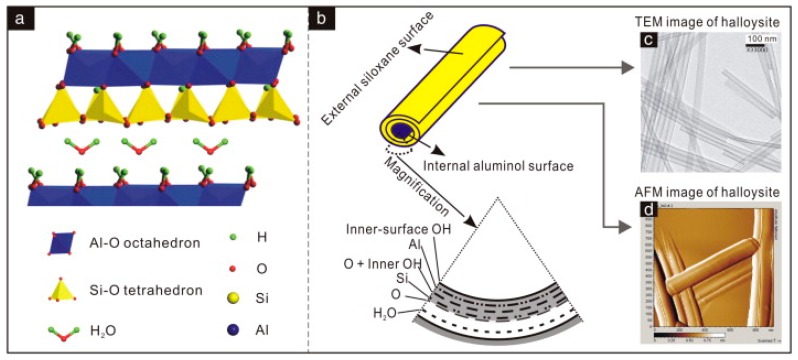
Schematic diagram of (**a**,**b**) the crystalline structure of halloysite; (**c**,**d**) Transmission electron and atomic force microscopy TEM and AFM images of halloysite. Reproduced with permission from [34], copyright by Elsevier 2015.

**Figure 1:**
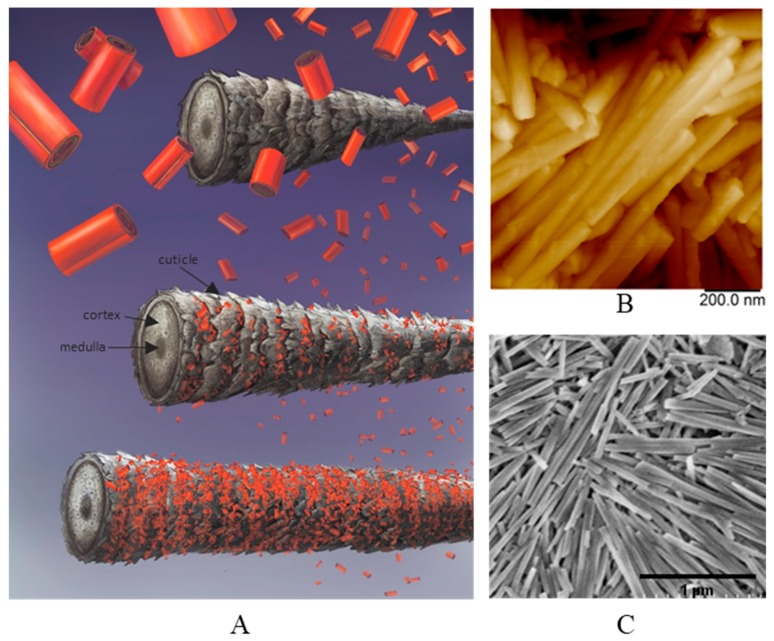
Introduction to the self-assembly of halloysite nanotubes on hair and development of the coating from anchoring in the cuticle to capillary force/drying driven surface assembly (**A**), and atomic force microscopic and scanning electron microscopic (AFM, SEM) images of dried clay nanotubes (**B**,**C**). Reproduced from Ref. [23] with permission from the Royal Society of Chemistry.

Halloysite clay nanotubes (HNT) are multiwall inorganic structures of 50–70 nm in outer diameter, 10–20 nm in inner diameter, and 500–1000 nm in length [24]. Belonging to the phyllosilicates (sheet silicates) group, these tubes are rolled aluminosilicate sheets, appearing as elongated cylinders, Scheme 1 [25]. Similar to many naturally formed minerals, water molecules are embedded between the wall sheets, imparting an empirical formula of (Al_2_Si_2_O_5_(OH)_4_ × nH_2_O) and an increased space between consecutive spiral layers from 0.7 to 1.0 nm [26]. The lumen volume can be increased by chemically etching alumina and widening the lumen diameter for higher dye/drug loading capacity [24]. The differing internal–external chemistry (Al_2_O_3_/SiO_2_) makes opposite inner/outer tubes’ charges at pH 4–8.5 (Figure 1). Such a structure makes the halloysite surfaces selective for charged molecules, increasing loading negative components into the tube’s lumens and positive ones at the outside surface [24]. The hydroxyl groups on the tube’s surface enable hydrophobization by silane grafting [26,27].

A variety of dyes and drugs can be loaded into the lumen, and the sustained release profile of the loaded compounds makes halloysite an efficient delivery material [28]. There is a diversity of compounds loaded into/onto halloysite and a controlled release, represented by tetracycline, ciprofloxacin, dexamethasone, nifedipine, paclitaxel, insulin [29], genetic material [30], and by its usage as bioscaffold for tissue engineering [31]. Adding their low toxicity, availability, and low price, HNTs appeared to be a competitive nanomaterial for biomedical research and applications [32,33].

#### 2.1.1. Cosmetic Applications

A recent study explored the potential of clay nanotubes for cosmetic applications through self-assembly. The technique combined selectivity of lumen loading, sustained release of active dyes, and the nanotubes’ self-assembly on the exterior hair surface [23]. The process takes advantage of the mesoporous structure of hair with 1 µm-thick flap-like cuticles covering the surface. A simple washing of hair for 3 min by 1 wt. % of water halloysite dispersion provides a 2–3 µm thick clay coating in and around the cuticle flaps. In an aqueous environment, the cuticles open up like flower petals and allow for the halloysite dispersion to enter the inter-cuticle space, where micro-confinement aligns the tubes under the influence of capillary forces during drying. Halloysite-encapsulated dye may be water soluble, or soluble only in organic solvents. Thus, the loading of the clay nanotubes may be performed from any, possibly harmful, solution, but when the formulation is competed, we apply these colored nanopigments to hair from safe aqueous dispersions.

**Figure 2:**
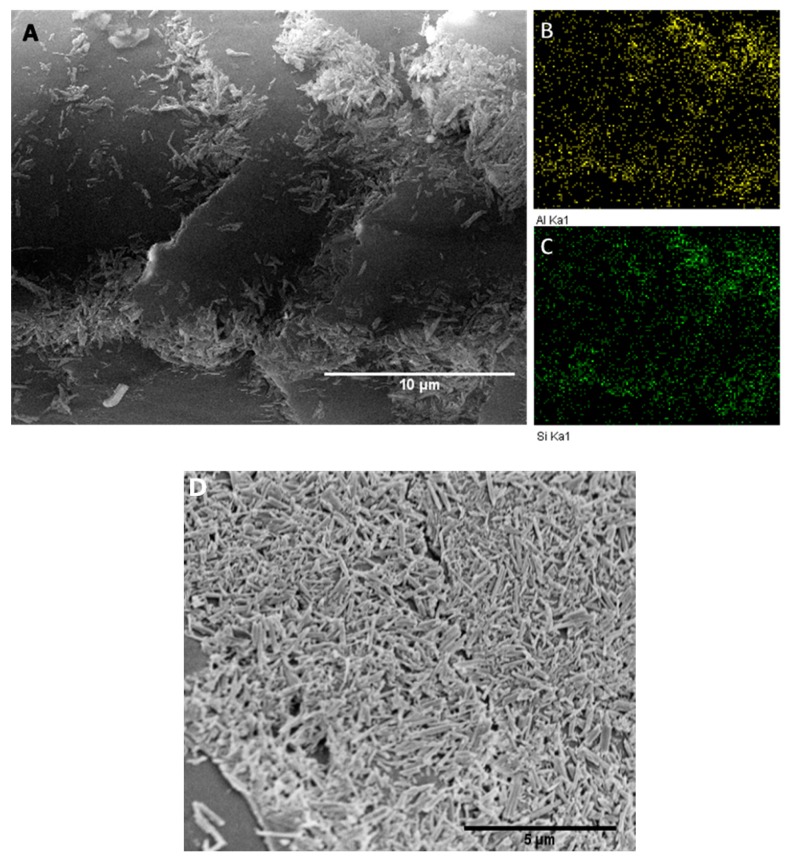
SEM image of halloysite coating in cuticle vicinity (**A**) and EDX mapping-aluminium (**B**) and silica (**C**) mapped of hair coated with halloysite. Al and Si areas represent a distribution of the nanotubes. (**D**) SEM image of hair completely coated with halloysite.

The choice of dyeing agent was demonstrated with 2-hydroxy-1,4-naphthoquinone, commonly known as lawsone. Lawsone is an unstable and insoluble extract of the popular henna plant (*Lawsonia inermis*). A crucial feature of the coating process is the selective modification of positively charged clay lumen by the adsorption of negative amphiphiles, which enable the encapsulation of hydrophobic agents such as lawsone. The 6.0 ± 1.0 wt.% of lawsone loaded into the halloysite lumen changed the color of old grey hair to bright vivid brown through the assembly process (Figure 1). Figure 2 shows that the coating originates from cuticles and is then spread over the whole hair (at very low HNT concentrations it may be restricted only to the cuticle area). The colored hair is able to stand as a semi-permanent dye, preserved for up to 10 shampoo washes. In particularly, a multiple shampoo with 10% sodium dodecyl sulfate gives a loss of the coloring clay nanopigment of ca. 30% after five and 50% after eight washing cycles.

The principle of natural dye loading into the halloysite lumen was extended to include the basic colors: blue indigo, purple alkanet extract, and yellow curcumin extract, for all spectra of hair color formulations. Our method, based on physical forces, is applicable over the spectrum of hair types and is less damaging and nontoxic to hair and skin.

**Figure 3:**
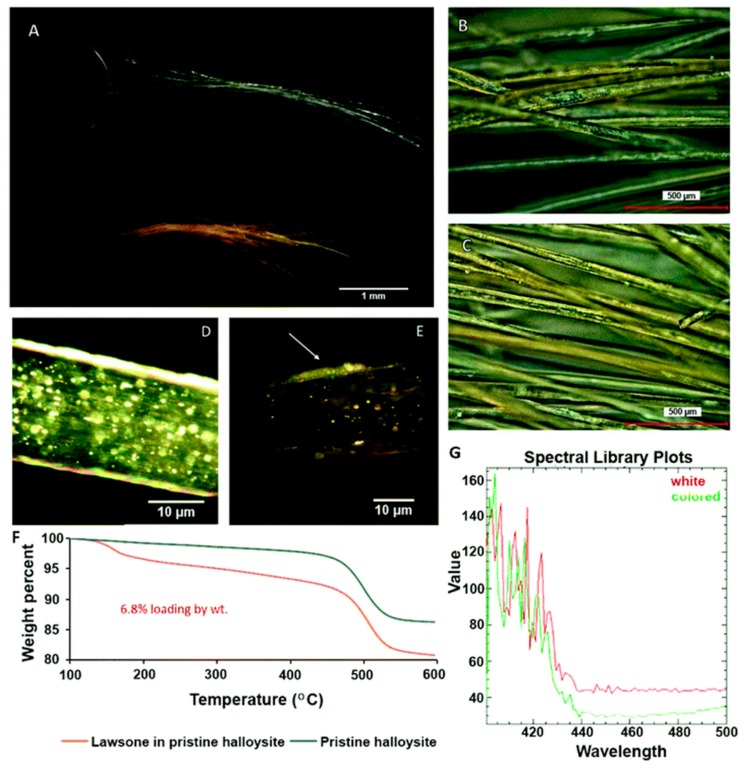
Color treatment of hair via halloysite self-assembly: grey hair (upper image, **A**) washed for 3 min and treated with an aqueous dispersion of lawsone-loaded halloysite gives a bright orange color (bottom image, (**A**)). High definition images of hair coated with brown lawsone loaded halloysite (**B**,**C**). Dark field images of intact grey (**D**) and colored grey (**E**) hair from the same person (note the color change and the visible halloysite-dye aggregate indicated by the arrow); reflected light spectra recorded in transmission dark field microscopy mode demonstrating the color change in grey hair after dye-halloysite composite deposition onto hair (**G**). Thermogravimetric analysis (TGA) results of lawsone-loaded halloysite tubes. (**F**) shows their increased weight loss against pristine halloysites referring to the incineration of organic dye content.

In Figure 3 shows images of grey human hair colored brown with the halloysite coating technique, using the nanotube loading with 6.7 wt.% of lawsone. Even though the halloysite loading is restricted by 10 vol.% of the lumen, this amount of dye is sufficient to color hair. Higher halloysite loading mans that some dye is adsorbed outside, which is not favorable because it may change physical-chemistry of the self-assembly process.

Typically, the leakage of dyes loaded into halloysite nanotubes occurs during 10–15 h, which much exceeds a possible time for hair exposure to water, and even this leaked dye will stay on hair, preserving color.

#### 2.1.2. Biomedical Applications

**Figure 4:**
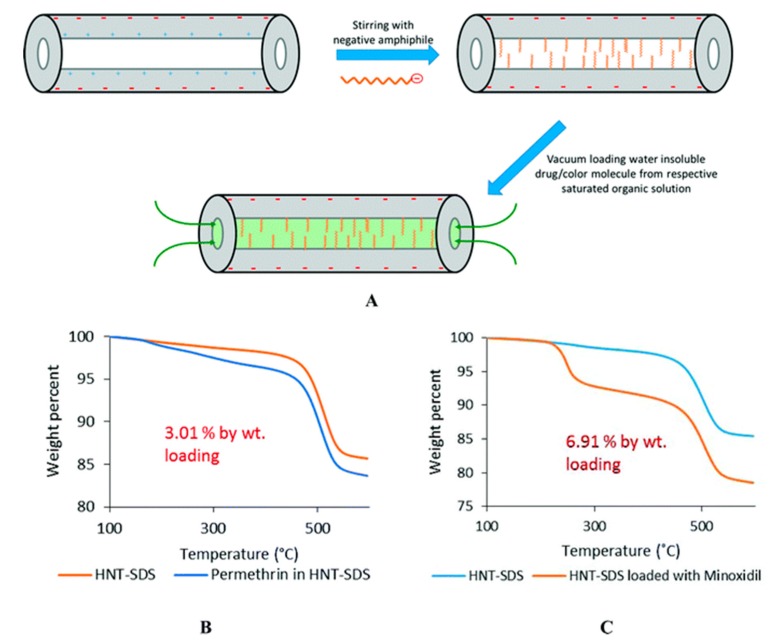
Illustration of sodium dodecyl sulphate (SDS) amphiphile coating inside the lumen, aiding in loading water insoluble compounds (permethrin and minoxidil) into the halloysite lumen (**A**). thermo-gravimetric (TGA) spectra of halloysites pre-treated with SDS and loaded with water insoluble active agents, permethrin (**B**) and minoxidil (**C**). Reproduced from Ref. [23] with permission from the Royal Society of Chemistry.

Anti-lice treatments to eliminate infestations provoked by the commonly-known human lice *Pediculus humanus capitis* are very important [35,36]. Resistance to common pesticides like pyrethroids and permethrin and re-infestations are the problems with conventional anti-lice formulations [37]. It is necessary for anti-lice drug delivery to be sustained and hair-localized. For effective anti-lice treatments, we loaded permethrin into the clay nanotube lumens. A lumen modification with negative amphiphiles (sodium dodecyl sulphate), to render the cavity hydrophobic and susceptible to encapsulate the highly insoluble permethrin, was developed, as shown in Figure 4 [23,38,39]. A slow and sustained release of permethrin from the tube’s lumens was demonstrated, elucidating the capabilities of this nanoclay-based anti-lice disinfestation strategy for a sustained hair treatment.

The permethrin-loaded nanoformulation was tested in vivo in nematodes, *Caenorhabditis elegans*, considered a convenient model organism [23,40]. Strong adhesion to the nematodes’ cuticle of the nanotubes, as well as their preferential intestinal presence, was shown. The results suggest the favored biocide effect of permethrin by its halloysite loading, perhaps due to nanotube intestinal penetration, leading to permethrin uptake enhancement, and achieving a worm death toll of 85% [23].

Another important issue regarding hair maintenance is hair loss treatments [41]. Since the introduction of topical minoxidil for androgenetic alopecia treatment, it has been included in several formulations for hair growth [42]. Researchers developed minoxidil-loaded nanoclay, to test the performance of the nanotubes. The loaded halloysites were prepared similarly to the permethrin-loaded ones, with methanol used as a solvent. The resulting halloysite formulations represent a strategy for topical hair surface coating with anti-hair loss agents, exhibiting a slow release profile. [23]

We have to underline that halloysite color formulation and drug loading may be used together, by the hair application of mixture of dyes and drug loaded nanotubes.

### 2.2. Carbon Nanotubes

Carbon materials, particularly graphene-based ones, have also shown promise for hair dyeing. Graphene-based sheets, obtained via exfoliation of graphite powders in the presence of strong oxidizing agents, are presented as graphene oxide and reduced graphene oxide. Graphene-based nanosheets were mixed with chitosan, yielding color nanoformulations. This demonstrated a toxic-free procedure for dyeing light-colored hair with dark shades, ranging from brown to black. These formulations exhibited a resistance to multiple shampoos and also antistatic and heat dissipation capabilities [19].

Chemically functionalized carbon nanotubes (CNT), synthetized for coloring hair, eyebrows, or eyelashes, usually follow well-defined steps: covering the target surface by a polymeric layer (amine, cationic or anionic), followed by contact with the chemically functionalized CNT for the first colorant layer. Additional repeated cycles allow for the formation of multiple intercalated layers, reinforcing the final color. Simple coating mechanisms, by dipping in CNT-dispersions, yielded a black color on bleached and non-bleached grey hair [43]. Peptide-based CNT colorants, formed by the coupling of a hair-binding peptide to the nanotube surface, formed diblock compositions and increased the affinity to hair by covalent conjugation [44]. Besides the biomedical and cosmetic context, the hair-related multifaceted applications of carbon nanotubes led to the development of CNT-based artificial fiber sensors [45]. A bioinspired artificial CNT hair sensor for air flow detection, with a “hair-plug” design, was composed of the nanotubes coated on micro-capillary support [46].

## 3. Nanotube-Based Formulations for Animal Hair Treatment

**Figure 5:**
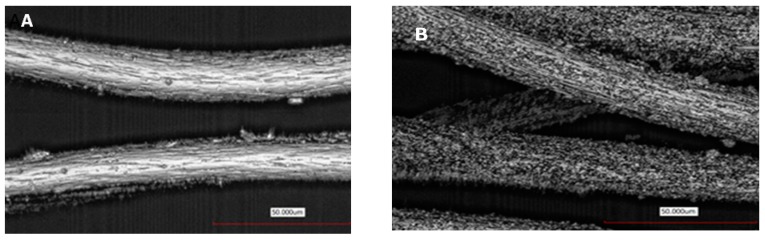
Wool fibers before (**A**) and after (**B**) halloysite dispersion exposure.

The indications for the design of veterinary treatments are similar to human hair. The majority of the physical and chemical properties remain consistent for hair belonging to different animal species. The clay nanotube assembly may be extendable to deliver veterinary drugs to animals, including dogs, cats, and sheep (Figure 5). The loading of biocidal compounds like permethrin creates avenues of anti-flea formulations for animals, especially those involved in farming activities [47].

**Figure 6:**
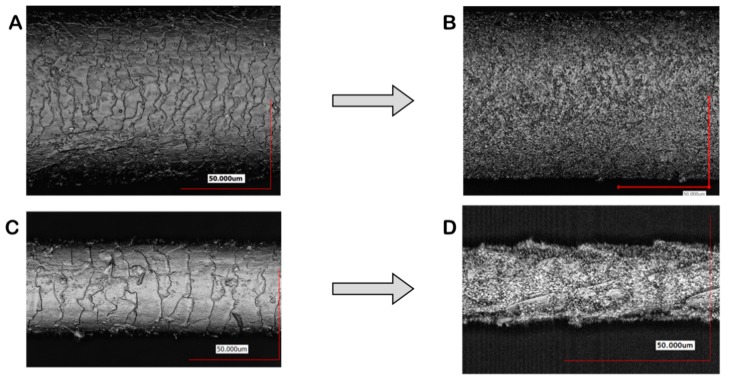

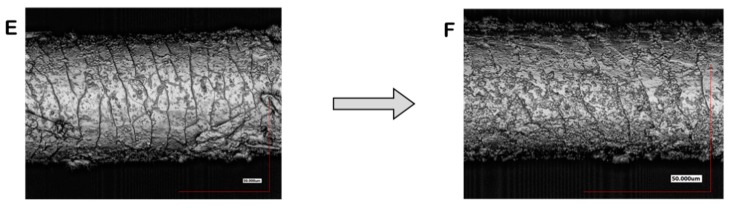
Clay nanotube coating for animal hairs. Bare and coated confocal microscopic images of dog (**A**,**B**), cat (**C**,**D**), and horse (**E**,**F**) hair.

Hair treatments applicable to human hair are transferable to animal hair as well. For example, the use of benzotriazole to protect the keratin composition of hair fibers from UV radiation was tested for animal care [48]. Similarly, polymer additives that prevent the deleterious effects of heat on hair are also indicated for veterinary care [49]. Figure 6 demonstrates successful halloysite nanotube coatings for dog, cat, and horse hair.

Both wool and hair have the same components—cuticle, consisting from overlapping scale cells of ca. 0.5 µm and enriched with cysteine, cortex and media [50]. The nano-assembly technique is based on physical forces and the specific structure of hair similar for hair and wool. The underlying phenomena of cuticle swelling and opening on wetting are similar for wool and human hair determining the main parameters of halloysite assembly for the both types of biological fibers.

## 4. Nanotubes’ Safety

Though nanomaterials are used in the cosmetics industry in an increasing fashion [51], concerns have been expressed regarding the potential toxicological profiles in contact with the human body [52]. The large surface area of nanoparticles can interfere with biological mechanisms [53]. Concerning the toxicity of nanotubes, similar to the other types of nanomaterials this depends on size, shape, and chemical composition. Halloysite safety was studied with worms, fish, and mice [40,54], subjected to oral administration of the nanotubes. Different aspects were measured, for instance, serum biochemical parameters, aluminum and silica contents in the liver, and oxidative stress measurements, by assessing the profiles of endogenous antioxidative enzymes, glutathione peroxidase, and superoxide dismutase.

In vivo halloysite toxicity testing in *C. elegans* by the ingestion of HNT-coated cells showed that, despite mechanical stress to the nematodes’ digestive system, no major harm was caused to the organism, eliciting little toxicity—contrasting with other nanomaterials, which can avidly experiment cellular uptake and transport to tissues. The experiment deemed halloysite as safe to soil nematodes, therefore suggesting that further industrial applications are probably safe for the environment [40]. Reduced uptake due to the length of the clay nanotubes and efficient excretion can be key aspects for the safety of this nanomaterial. Another study showed halloysite to be non-toxic for cells; for that, the nanotubes were added to different cell lines for cytotoxicity assessment. Cellular viability was determined and was shown to be safeguarded until concentrations up to 0.1 mg/mL. Moreover, laser confocal microscopy provided information regarding cellular uptake, in which halloysites were preferentially localized in the nucleus vicinity [55].

The oral administration of halloysite had a growth stimulatory effect in mice, with no hepatic toxicity (for the low dose), but had an opposite effect in mice administered with the high dose. In addition, an induction of aluminum ion accumulation was observed, and thus oxidative damage in the liver, leading to hepatic dysfunction and histopathological modifications, despite no silica ion accumulation being verified [54].

The inhaled clay nanotubes suggested not only subchronic toxicity in mice, but also autophagy blocking abilities, with a consequent accumulation of sequestosome-1, a ubiquitin-binding protein, resulting in exaggerated apoptosis, anti-flame responses, and oxidative stress generation [56]. They also could be an effective way of diminishing HNT-mediated inhaled toxicity via a p62 clearance enhancement mechanism. The cosmetic ingredient review (CIR) panel concluded that aluminum silicate, as well as other minerals, was safe for cosmetic use, despite emitting a reservation regarding the need to minimize inhalation of these ingredients [57].

Carbon nanotubes have raised health concerns, as several in vitro and in vivo studies have underlined. Results suggest that the relevance of physicochemical characteristics, such as size—shorter tubes have proven to be less toxic when compared to the longer ones—shape, length, and functionalization, which can affect bio-distribution and elimination, hence contributing to higher or lower potential toxic responses [58]. Pulmonary exposure may be the most probable route of exposure to carbon nanotubes, but in the occupational work context. This way, evidence shows that a carbon nano-fibrous shape is important for the development of lung pathogenicity, including inflammation, fibrosis, and granuloma formations [59].

## 5. Future Prospects

The relevance of hair follicle-targeted drug delivery is a significant goal. Since follicular-targeted stem cells have shown excellent results in hair regrowth, nanoparticles can be used as a non-viral method to deliver and assist the regeneration of induced pluripotent stem cells via the transfection of a gene, aiming to circumvent the pathological condition [60]. Other studies gravitate around in vitro models for studying de novo hair follicle regeneration and treatment [61].

With HNT-encapsulation, we are opening the way for the application of water insoluble dyes, which prior to this were not used in hair coloring. For this, one could dissolve such dyes in an organic solvent for the loading process, and the resulting nanopigments eliminate traces of the solvent. In many cases, such loading may request hydrophobization of the clay nanotube lumens, as demonstrated in [38,39]. For applications, such tubule nanopigments may be dispersed in water, resulting in stable long-standing coloring.

New approaches for dye/drug based nanosystems are developing, including additional care with encapsulated keratin, curcumin, and other vitamins. Similar to other nanomaterials, Halloysite and carbon nanotubes were exploited regarding their suitability for dye-loading and hair delivery intents or solely nanotubes themselves. These new avenues are promising to open new nanotube-based formulations for hair dye/drug delivery, with increased efficiency and enhanced biosafety.

## 6. Conclusions

Although haircare products, including both hair treatment and hair embellishment formulations, have been widely developed, a need for innovation is rising with an increasing demand and requests to make the coloring process less irritating. Nanotechnology may change a panorama of cosmetic and biomedical sciences because it makes it possible to avoid a paradox in the process of hair coloring, when one is trying to use an aqueous dye solution to get not-water soluble hair coloring, which inevitably needs a chemical reaction during coloring. The encasing of dye or drugs into nanoparticle containers allows the development of stability in the water pigment (like color-loaded clay nanotubes), which then could be applied onto hair to form aqueous dispersions.
